# Sexual Dimorphism of NAFLD in Adults. Focus on Clinical Aspects and Implications for Practice and Translational Research

**DOI:** 10.3390/jcm9051278

**Published:** 2020-04-28

**Authors:** Amedeo Lonardo, Ayako Suzuki

**Affiliations:** 1 Operating Unit Metabolic Syndrome, Azienda Ospedaliero-Universitaria di Modena, Ospedale Civile di Baggiovara, 41126 Baggiovara MO, Italy; 2 Division of Gastroenterology, Durham VA Medical Center and Duke University Medical Center, Durham, NC 27705, USA; ayako.suzuki@duke.edu

**Keywords:** cardiovascular risk, clinical course, cirrhosis, diabetes, epidemiology, HCC, NASH, pathogenesis, sex medicine, steatosis, sex differences, serum uric acid, type 2 diabetes, tumors

## Abstract

Nonalcoholic fatty liver disease (NAFLD) embraces the clinico-pathological consequences of hepatic lipotoxicity and is a major public health problem globally. Sexual dimorphism is a definite feature of most human diseases but, under this aspect, NAFLD lags behind other medical fields. Here, we aim at summarizing and critically discussing the most prominent sex differences and gaps in NAFLD in humans, with emphasis on those aspects which are relevant for clinical practice and translational research. Sexual dimorphism of NAFLD is covered with references to the following areas: disease prevalence and risk factors, pathophysiology, comorbidities, natural course and complications. Finally, we also discuss selected gender differences and whether sex-specific lifestyle changes should be adopted to contrast NAFLD in men and women.

## 1. Introduction

### 1.1. Nonalcoholic Fatty Liver Disease (NAFLD) Definitions and Burden

Nonalcoholic fatty liver disease (NAFLD) describes the large spectrum of hepatic and extra-hepatic pathophysiological and clinical manifestations, which result from the liver becoming an ectopic deposit of fatty substrates [1]. NAFLD covers the clinico-pathological gamut including uncomplicated steatosis, steatohepatitis, which is steatosis accompanied by hepatocyte damage and inflammation, cirrhosis and hepatocellular carcinoma (HCC). All of them occur, in mutual and bi-directional association with the Metabolic Syndrome (MetS) and in the absence of competing causes of liver disease [2]. Owing to its worldwide epidemiological diffusion, protean features and impaired life expectancy and quality of life, NAFLD poses an enormous burden on global society which is also projected to rise in the forthcoming years [3,4,5]. By affecting a quarter of the world’s general population, NAFLD prevalence became quite substantial on a global basis [6]. More than 64 million individuals in the USA, and ~52 million people in the four most populated European countries are estimated to have NAFLD, accounting for a yearly burden of direct medical costs of about 103 billion dollars in the USA and approximately 35 billion euros in Europe [4]. Costs are highest in the 45–65-year-old age group, and further increased by including societal costs [4].

### 1.2. Sex Medicine

Human diseases, such as cardiovascular diseases, may commonly exhibit a pronounced sexual dimorphism, i.e., significant differences in the pathophysiology, epidemiology and clinical outcomes between men and women [7].

“Sex differences” identify biological differences between men and women, whereas “gender differences” define those socio-cultural variations (e.g., norms, roles and relations) between men and women, which are formed through the interaction with a culture and society [7].

Since the 1980’s, the National Institutes of Health (NIH) have been reinforcing the significance of examining sex/gender differences in research. After 1990, multiple organizations evolved globally to foster and facilitate such efforts across different disciplines. More recently, NIH issued a policy in 2014 for all federal funding applicants to include both male and female animals in preclinical studies. These efforts resulted in an escalating number of publications addressing sex differences across disciplines and fields that are relevant to NAFLD, such as diabetes, coronary artery disease and immunology [8,9].

### 1.3. Sex Differences in NAFLD and Aim of This Review

However, compared to these other areas of medicine, our understanding of sex-differences in NAFLD, owing to inexplicable reasons, has remained sensibly more limited so far [9]. On this background, our aim was to summarize and critically discuss the clinically relevant features characterizing sex differences in NAFLD in adults. Experimental studies provide consistent clues as to the presence of sexual dimorphism of NAFLD in animal models and cell studies (Table 1) [10,11,12,13,14,15] and references to these models will shortly be made here whenever human studies are lacking. Nevertheless, the specific scope of the present review article is to develop those aspects which either already have or may potentially have a relevance for clinical practice and translational research over the short run.

### 1.4. Research Strategy

In order to retrieve the pertinent bibliographic material, we searched the PubMed database using the following keywords: “steatosis” OR “fatty liver” AND “sex” OR “gender” OR “sex differences”. Priority was given to data published in humans and in core clinical journals over the last five years. In addition, particular care was exercised in checking cross-references relevant to address the questions indicated by the paragraphs and sub-paragraphs of our review article.

## 2. Epidemiology

### 2.1. General Population Studies

A detailed analysis of epidemiological features of NAFLD as emerging in *population* studies has recently been published elsewhere [9]. In short, data consistently indicate that the overall prevalence of NAFLD is higher in men than in women [16,17,18,19,20,21,22,23], and that male sex [24] and menopause [25] are specific risk factors for NAFLD. As a result, the prevalence of NAFLD is similar in men and in women after the age of 50–60 years [16,26].

As regards the relationship of sex with the development of fibrosis in general-population studies, data are conflicting in as much as they indicate no sex difference [23,27,28], or fibrosis as more prevalent in males when assessed with either (fibrosis 4) Fib4 [27] or liver stiffness [29]. Most of these studies have assessed sex/gender differences in fibrosis progression without considering menopausal status, which may partly explain the inconsistent results.

### 2.2. Studies Based on Clinical Populations

A growing body of the literature exists in clinical observational studies of NAFLD. These findings should be interpreted with extra caution as the study populations were defined in clinical settings and are subject to selection biases, although the utilization of clinical and histological data allows accurate diagnosis and precise disease staging. Despite the pitfalls, studies conducted in clinical cohorts may have significant implications to similar clinical populations and provide insights and research questions to be further tested in future research.

The analysis of clinical observational studies indicates that the prevalence of NAFLD is up to three-fold higher in men than in women and that sex-specific differences exist in relation to age [17,30]. In parallel, male sex has been associated with incident NAFLD independently of age and metabolic factors [26,30]. A Korean study found that, compared to non-obese controls, non-obese individuals with NAFLD were older and predominantly male, had higher body mass index (BMI) and larger waist circumferences, further to additional components of the MetS [31]. Non-obese subjects with NAFLD included a relatively higher proportion of women compared to obese subjects with NAFLD (10.3% versus 3.5%). A Chinese study found that female sex was an independent risk factor of insulin resistance in those with NAFLD, although it has to be noted that the study did not consider menopausal status in the analysis [32]. Collectively, the studies discussed above may suggest potential intersections between race/ethnicity, age and sex/gender in NAFLD. Further studies considering the key risk modulators (i.e., age, sex/gender, menopause and race/ethnicity) are warranted.

**Figure 1:**
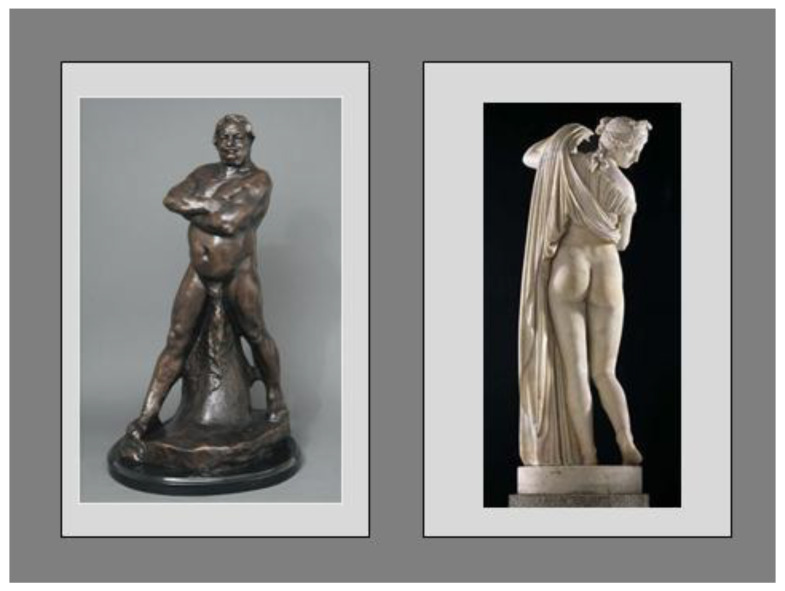
The android- and gynoid-type distribution of adipose tissue depots is the anatomical substrate predisposing to or protecting from metabolic disease. Left-hand side Panel—*Balzac Nude Study* by Auguste Rodin (1891). Although, in the past, the physiognomy of body fat distribution represented above may possibly have inspired a sense of importance and authority, we now know that it is the typical phenotype associated with MetS and its various complications. Following weight loss, men experience greater reduction in visceral fat and potentially greater improvements in metabolic profile than women, despite similar levels of weight loss [36]. Right-hand side Panel—*Callipygian Venus* (first or second century BC), a roman copy of an older Greek original (Ἀφροδίτη Καλλίπυγος, i.e., “Aphrodite/Venus of the beautiful buttocks”).

Evidence suggests that NAFLD has different risk factors in adult men and women [30,33], and in boys and girls [34]. In a Japanese study, waist circumference was negatively correlated with liver/spleen density ratio by computer tomography only in men [35], although visceral adiposity was negatively correlated with liver/spleen density ratio in both genders, which is probably explained by a higher ratio of subcutaneous abdominal adiposity versus visceral adiposity in women, indicating that waist circumference may not be an accurate risk measure for women. Endogenous gonadal hormones play a major role in the development of NAFLD via altered body fat distribution (Figure 1) [36,37,38] and through their action on metabolics.

The marble statue in Figure 1 depicts a typically gluteo-femoral distribution of body fat which identifies female beauty, youth/fertility and good metabolic health. It may even play a role in determining overall life expectancy, which is more elevated in women than in men owing to subcutaneous white adipose tissue being the main source of adiponectin [37,38], contributing to metabolic homeostasis in reproductive women [39]. These data accentuate the importance of considering reproductive lifecycle and health in the clinical research of NAFLD while stressing the notion that a variable of menopausal status (or age surrogate) must be analyzed and documented whenever addressing differences between men and women. Asian studies found that the NAFLD prevalence was higher in men than in women under the age of 50 years but became higher in women aged over 50 years [22]. Among men with NAFLD, a low serum testosterone level was associated with a higher likelihood of having nonalcoholic steatohepatitis (NASH) and severe fibrosis [40].

Whether the risk of NASH and advanced fibrosis is definitely higher in women than in men remains controversial, with some studies supporting this contention and others rejecting it (Table 2) [41,42,43,44,45,46,47,48]. A recent review by Tobari and Hashimoto delivered an update on the features of NAFLD, focusing on the effects of key modifiers of disease such as age, sex and BMI [49]. The authors highlight the inconsistent findings published in the literature on sex differences in NASH fibrosis. A previous study showed that men had a higher risk of more severe fibrosis compared to pre-menopausal women, while the severity of liver fibrosis was similar between post-menopausal women and men at a given level of hepatic necro-inflammation [46]. In their own study, Yatsuji et al. had observed NASH to occur rarely among young women, while (probably post-menopausal) women were predominant in the >55-year age group [47]. Labenz et al. evaluated the independent predictors of fibrosis in biopsy proven NAFLD by studying 261 patients with non-cirrhotic NAFLD. The prevalence of stage 3 fibrosis was 15.7% and the independent predictors of advanced fibrosis were type 2 diabetes (T2D) and arterial hypertension, rather than sex [48].

It must be pinpointed that the majority of the studies did not clearly distinguish pre- versus post-menopausal women in their analyses, which may at least partly explain the inconsistent findings on sex differences. Several recent studies in which menopausal status was considered in the analysis found that men, compared to pre-menopausal women, have a higher risk of advanced fibrosis while after menopause, both sexes display a similar severity of liver fibrosis, suggesting that estrogen protects from the development of fibrosis [46,50,51]. Finally, NASH has been independently associated with the male sex in a study evaluating morbidly obese individuals (58 men aged 41 ± 8.6 years and 307 women aged 40.9 ± 8.9 years) undergoing gastric bypass [52]. No data from prospective or retrospective cohort studies are available to address the impacts of sex and menopause on the progression of NASH fibrosis. Collectively, these data suggest that age and sex interact in the development of NAFLD and the histological severity of NAFLD fibrosis. 

## 3. Key Factors Contributing to Sex Differences in NAFLD

Owing to evolutionary pressure, biological sex is a major determinant of body composition, energy storage and partitioning. It is widely believed that, as a result of ancestral adaptations that aim at preserving their role during pregnancy and lactation, mammalian females are equipped to better withstand phases of under-nutrition [53]. This will account for women’s predisposition to store fat in the subcutaneous adipose tissue (a site which is particularly suited for large and long-term storage), whereas men (who tend to store fat in the visceral adipose tissue) are deemed to be more inclined to mobilize their fat stores to sustain physical activity [53]. These physiological and evolutionary grounds mirror themselves into physio-pathological and clinical counterparts. For example, compared to men and post-menopausal women, women of fertile age are typically spared from storing fat in the visceral and ectopic compartments, are more insulin-sensitive and display a higher capacity to secrete insulin and incretin, being relatively spared from the development of T2D and NAFLD [30,53]. Sexual dimorphism in lipid metabolism is well-preserved in mammals over a long evolutionary period [54]. Given such well-known sex differences in metabolisms, which is the key mechanism in the NAFLD pathogenesis, it is critical to decipher the biological bases underlying sex-specific metabolic features in order to better pursue precision medicine in the NAFLD field. Furthermore, a recent multi-omics integrative approach (i.e., Mergeomics) discloses sex- and tissue-specific genes, processes and networks accounting for sex differences in NAFLD, supporting the notion of developing sex-specific precision medicine. Using the HMDP and high-fat and high-sucrose diets, Kurt et al. integratively evaluated genotypes of the mice in the panel and genetic expression in the liver and adipose tissue, and correlated them with hepatic triglyceride content in both sexes and predicted sex- and tissue-specific pathways perturbed in NAFLD [13]. The authors demonstrated that, certain ‘processes’ (such as adaptive immunity, metabolism of branched chain amino acid, oxidative phosphorylation and cell cycle/apoptosis) are perturbed in NAFLD in both sexes, although identified perturbations in NAFLD often differ between sexes and/or the tissues. They also identified sex-specific perturbations (e.g., metabolism of vitamins and cofactors and ion channel transport for female only and metabolism of phosphatidylinositol and insulin signaling for male only) [13]. The study did not distinguish effects of innate sex differences versus sex hormones, but, regardless, these findings reiterate the significance of studying both males and females in experiments and consider possible disparities in designing the studies.

In this section, we shall analytically address specific roles of sex chromosomes and sex hormones on metabolic features and NAFLD, discuss sexually dimorphic contributing factors to NAFLD, organ-specific insulin resistance, aging and obesity, and also briefly address the interesting interplay between metabolic features and race.

### 3.1. Sex Chromosomes

Although sexual dimorphism in metabolic disorders may intuitively be attributed to the biological effects of gonadal hormones, the set of sex chromosomes may also play a role [55]. Data from the four core genotypes mouse model have revealed that X chromosome dosage (i.e., XX as compared to XY genotypes), independently of gonadal sex, plays a role primarily in feeding behavior, which in turn determines adiposity and its adverse metabolic correlates, i.e., fatty liver, altered glucose homeostasis and serum lipid profile [55,56]. This notion may more easily be appreciated when considering that the gynoid pattern of fat storage in women serves to provide energetic resources to sustain their reproductive role [56]. Differential gene dosage of X chromosome genes that escape from inactivation and distinct genomic imprints on X chromosomes inherited from either the mother or father are implicated in affecting the risk of metabolic disorders [55].

Men with Klinefelter Syndrome (XXY) are associated with an increased prevalence of insulin resistance, T2D [56] and NAFLD [1]. Women with Turner syndrome, especially ones with 45X monosomy, are also associated with an increased risk of metabolic disorders [57]. These clinical associations support the importance of sex chromosomes in metabolic regulation and the risk of metabolic disorders. Whether Klinefelter syndrome and Turner syndrome present an increased risk of NAFLD progression remains unknown and requires further investigation.

Studies on bio-physiological changes observed in transgender individuals provides important insights pertaining to metabolic regulations by sex hormones in each sex. A recent longitudinal analysis in transgender individuals revealed significant changes in metabolic profiles both in transgender women and men [58], suggesting potential implications to their NAFLD risk. No data are currently available for assessing the risk and clinical outcomes of NAFLD in transgender individuals.

### 3.2. Sex Hormones

Overwhelming evidence from experiments suggests that sex hormones affect mechanisms that play pivotal roles in the NAFLD development and progression: metabolisms, oxidative stress, cellular survival/death, immune response and tissue repair. The clinical implication of these findings awaits future clinical and translational studies [13].

Analysis of NAFLD and NASH secondary to hypogonadism supports the notion that both estrogens and androgens play significant roles in maintaining metabolic health. There is an intriguing interaction to be noted: excess of androgens is associated with enhanced risk of NAFLD in women while androgenic deficiency is associated with an increased risk of NAFLD in men [1]. Therefore, the significance and effects of sex hormones on NAFLD may differ between men and women, which needs to be considered in designing studies.

In male rodents, protection against NAFLD and insulin resistance is exerted by androgens by activating both androgen and estrogen receptors (ERs) (the latter occurs after conversion of testosterone to estradiol) [59]. Similarly, testosterone deficiency in men, such as occurs in hypogonadism, also carries an excess risk of NAFLD [1]. Conversely, in female rodents, androgens promote NAFLD and dysglycemia, whereas estradiol protects from NAFLD [59]. In post-menopausal women, the prevalence of NAFLD is higher than pre-menopausal women owing to declining estrogen levels, which carries an increased risk of gaining weight, visceral redistribution of body fat and dyslipidemia [60]. Many facets of sex differences in the risk of coronary artery disease are affected by the sexually dimorphic activities of estrogens in the muscle, adipose tissue and the liver. In humans, a predominant form of circulating estrogen is 17β estradiol (E2), the signaling of which occurs via activation of the two ERs, alpha (ER-α) and beta (ER-β), plus the G-protein coupled Estrogen Receptor (GPER) [61]. Among the human hepatic genes, as many as 1000 are differently expressed in men and women, notably including genes controlling lipid metabolism and cardiovascular risk. Many of these genes display variation depending on estrus cycling in the mouse. Future directions likely rely on targeting estrogens to specific tissues, or specific aspects of the signaling pathways in order to recapitulate the protective physiology of pre-menopause and potential therapeutics after menopause [61]. The specific actions of E2 on lipid metabolism accounting for women of fertile age exhibiting protection from NAFLD as well as the implications of differences among mice and humans in estrogen-regulated liver lipid metabolism have extensively been covered by Palmisano [61].

### 3.3. Insulin Resistance

Insulin resistance is one of the most significant drivers in the development and progression of NAFLD. Consistent with the concept of “metaflammation” (i.e., metabolic as opposed to infectious, low-grade, systemic inflammation), insulin resistance, together with altered gut microbiota, unbalanced adipokines, lipid-induced endoplasmic reticulum stress, mitochondrial dysfunction and increased oxidative stress, set the stage for steatogenesis and hepatic fibrogenesis [2,62]. The metabolic and molecular pathways accounting for these phenomena involve increased availability of substrates such as glucose (owing to hepatic insulin resistance) and fatty acids (resulting from adipose tissue insulin resistance) while activating those transcription factors: Carbohydrate Response Element Binding Protein (ChREBP) and Sterol Response Element Binding Protein 1c (SREBP-1c), in the liver. The activated transcription factors then promote de novo lipogenesis and, by amplifying the triglyceride synthesis and inhibiting intra-mitochondrial beta-oxidation and export of these from the hepatocyte into the bloodstream, trigger an inflammatory reaction and stellate cell activation [63,64,65,66,67,68,69,70,71,72,73]. On these grounds, a cause-and-effect relationship links insulin resistance with steatosis [74]. For example, animal models support a direct causal association between insulin resistance, hyperinsulinemia and hepatic steatosis [75]. Patients with mutations in protein kinase B (AKT)2 exhibit strong resistance to insulin’s glucoregulatory actions while retaining the sensitivity to insulin’s lipogenic effects [76]. Additionally, steatosis specifically occurs in those hepatocytes surrounding metastatic insulinomas and transplanted islet cells [77]. Finally, certain insulin-sensitizing lifestyle changes (e.g., weight loss) and pharmacological interventions (such as the thiazolidinediones, biguanides, glucagon-like peptide-1 receptor agonists and the incretins) lead to decreased liver fat content by improving insulin resistance [78]. Not only is insulin resistance directly involved in the NAFLD development but it is also involved in the fibrotic progression of NASH. Rosso et al. demonstrated that oral glucose insulin sensitivity index (OGIS) was associated with peripheral insulin sensitivity and inversely associated with an increased risk of significant/advanced liver damage in non-diabetic subjects with NAFLD. Indeed, OGIS predicted advanced liver fibrosis better than NAFLD fibrosis score and was also able to discriminate F2 from F3/F4 [79]. Fujii et al., by evaluating a large cohort of non-diabetic patients with biopsy-proven NAFLD, found that, in the exploration cohort, age, dyslipidemia and HOMA-IR were the independent predictors of advanced fibrosis; however, only age and HOMA-IR independently predicted advanced fibrosis in the validation cohort [80]. More recently, it has been appreciated that the association of NAFLD (and NASH) with insulin resistance (and therefore T2D and the MetS) is complex and that a mutual, bi-directional association exists between NAFLD and insulin resistance [81,82,83,84,85,86].

Men and women exhibit significant differences in the risk of cardiovascular diseases [87], type 2 diabetes [88], NAFLD [30] and HCC [89,90]. Could this occur owing to biological or socio-cultural differences (i.e., sex differences versus gender differences)? While a definite answer to this question is not readily available, several sex/gender-related differences in energetic homeostasis should be highlighted: organ-specific insulin resistance, potential intersection with ethnicity and responses to fasting, hypoglycemia, exercise, obesity and aging.

#### 3.3.1. Muscle

As regards muscle insulin sensitivity, compared to men (*n* = 23, age 24 ± 1 years), young women (*n* = 15, aged 27 ± 2 years) are generally more insulin-sensitive in skeletal muscle and have higher serum concentrations of adiponectin [91]. Adiponectin is an anti-inflammatory, anti-steatotic, insulin-sensitizing adipokine. Studies show that large differences exist between men and women in a serum adiponectin level and the adiponectin regulation of insulin-dependent glucose uptake in skeletal muscle. A lesser muscular sensitivity to adiponectin in women may be accounted for by a lower skeletal muscle AdipoR1 protein expression and a lower expression of adiponectin-sensitive type 2 muscle fibers in women than in men [91,92]. Women have greater insulin sensitivity than men at the whole-body level, which is largely attributed to greater glucose uptake by skeletal muscle [93]. Similarly, a lower fasting glucose level and a greater postprandial glucose disappearance rate are observed in elderly women than in men [94]. Muscle mitochondrial adenosine triphophate (ATP) production, which is in general directly associated with muscle insulin sensitivity, is reportedly lower in women than in men, posing intriguing questions pertaining to sex-specific mechanisms in energy homeostasis [95].

#### 3.3.2. Liver

The development of hepatic insulin resistance is a key pathological step to drive atherogenic dyslipidemia, the risk of T2D and cardiovascular diseases in those with obesity and NAFLD [96,97]. Estrogens play significant protective roles in hepatic insulin resistance, not only in women but also in men. The significance of the estrogen signaling, via estrogen receptor-α (ERα), has been shown in men and male animals in preventing obesity and insulin resistance [98,99,100]. Consistent with this paradigm, estrogen treatment reverses pathway-selective insulin resistance in ovariectomized animals by promoting the activity of insulin on glucose metabolism and limiting hepatic steatogenesis [101]. However, the risks and benefits of hormone replacement therapy among post-menopausal women with NAFLD are not fully investigated, and are pending future investigation.

#### 3.3.3. Adipose Tissue

As regards insulin resistance in the adipose tissue, a recent study conducted in a non-obese population in Singapore found that, compared to men (*n* = 32; BMI 21.8 ± 1.5 kg/m^2^; age 42 ± 14 years), women (*n* = 28, BMI 21.4 ± 2.0 kg/m^2^; age 41 ± 13 years) have a more insulin-sensitive adipose tissue as assessed with mixed meal-induced suppression of non-esterified fatty acids NEFA concentrations; however, this superior insulin sensitivity in women is not observed in skeletal muscle, suggesting that sex differences in glucose tolerance probably result from sexually dimorphic hepatic insulin action [102]. In contrast, among older individuals across the body weight spectrum, visceral adipose tissue is more strongly associated with cardio-metabolic risk in women than in men. Gill et al. quantified pericardial adipose tissue (PAT) and abdominal adipose tissue using computed tomography (CT) scans in 151 women and 152 men (mean age = 57 ± 17 years). Compared to lean individuals, PAT was higher in overweight and obese individuals and in men than women. The association of PAT with BMI, abdominal fat, fasting glucose and serum lipids was stronger in women than in men [103].

#### 3.3.4. Ethnicity

Ethnicity, a definite risk factor for NAFLD, may also be envisaged under a pathophysiological perspective pertaining to sex/gender-related insulin resistance (i.e., sex/gender-race/ethnicity intersection). For example, Sumner et al. evaluated 127 nondiabetic African-Americans (mean age 32 ± 4 years, classified into three groups: non-obese (31 men, 24 women), obese (17 men, 14 women) and severely obese (12 men, 29 women) based on BMIs) with anthropometric measurements, fasting triglyceridemia, suppression of free fatty acid (FFA) during oral glucose tolerance test (OGTT) and 2 h euglycemic-hyperinsulinemic clamp. Data have shown that both obese African-American men and women are resistant to the glucoregulatory action of insulin, compared to non-obese subjects. Interestingly, obese African-American men, but not women, are resistant to the anti-lipolytic action of insulin, which may contribute to the higher prevalence of obesity, and ultimately type 2 diabetes, among African-American women [104]. Sex difference in the effect of obesity on insulin’s antilipolytic action was not observed in a Caucasian population. Both obese Caucasian men and women are resistant to insulin’s antilipolytic action [105], suggesting a sex/race intersection in the effect of obesity on insulin’s action.

#### 3.3.5. Stressful Situations: Fasting, Hypoglycemia, Exercise and Post-Prandial Lipidemia

In both sexes, fasting-hypoglycemia-exercise conditions will trigger homeostatic (autonomic, neuroendocrine and metabolic) physiologic responses to restore euglycemia through glycogenolysis, proteolysis and lipolysis, which collectively supply substrates (lactate, pyruvate, amino acids and glycerol) and energy (FFA) for gluconeogenesis to occur. Further to these general responses, there are sex-specific differences in substrate utilization under such physiologically stressing events, women have greater reliance on lipid metabolism while men preferentially utilize carbohydrates [106].

Fasting induces increased serum concentrations of fatty acids and ketones, associated with declining serum glucose levels both in men and in women [107]. However, these homeostatic responses are significantly more pronounced in women [106]. During fasting, compared to men, women have lower plasma glucose levels despite higher plasma FFA, suggesting protection from FFA-induced insulin resistance [108,109]. The mechanisms underlying this more efficient glucose disposal in women than in men are not precisely characterized. However, they are deemed to be associated, at least in part, with women being spared from the insulin-desensitizing effects of myocellular accumulation of ceramide [110], which protects them from FFA-induced insulin resistance [109].

Cell membrane proteins involved in the transport of long-chain fatty acids (e.g., fatty acid binding protein and fatty acid translocase (FAT)/CD36) may account for sex differences in diet and exercise [111]. An experimental study conducted in rats found that whereas levels of the major fatty acid translocase in skeletal muscle (CD36/FAT) were similar in male and female animals at baseline, male rats—but not females—exhibited a striking 50% decrease in CD36/FAT protein content following lipid infusion [112]. This finding may reasonably suggest that the difference in CD36/FAT protein levels in skeletal muscle following lipid infusion may account for the enhanced FFA clearance and the protection from fat-induced insulin resistance in female rats, through diverting fatty acids to a metabolic pathway that does not impair the transmission of insulin signaling [112]. Similar findings have been reported in the rat and human liver following stimulation with Growth Hormone [113]. Additionally, circulating estrogens exerting beneficial metabolic effects pre-menopause [114] and sex differences in the composition of muscle fiber could also be a factor [108].

Compared to males, in females, hypoglycemia results in a significantly reduced counter-regulatory response (i.e., lower epinephrine, norepinephrine, glucagon, growth hormone, pancreatic polypeptide and hepatic glucose production responses) [106].

Sexual dimorphism also exists in physiology during exercise and postprandial lipemia: higher glycerol and FFA responses in women compared to men while higher carbohydrate oxidation rate in men during exercise. Several explanations have been put forward to account for these sex differences: (a) dimorphism in the distribution of body fat, (b) estrogens affecting circulating catecholamines, (c) sex differences in the sensitivity to epinephrine and (d) differences in central nervous system driving sex-specific neuroendocrine, autonomic and metabolic responses [106]. As regards postprandial lipidemia, women, compared to men, exhibit lower incremental areas under the curve for postprandial triglyceridemia [115].

The strategy of energy partitioning by maintaining lipid synthesis under stressful conditions using amino acids as an energetic source is a key sex difference for the hepatic metabolism. Della Torre et al., by extensively applying metabolomics and transcriptomics to a mouse model, have demonstrated that, following short-term fasting, mediated by the estrogen receptor α in the liver, females preserve the biosynthesis of lipids utilizing aminoacidic precursors, and male animals instead slow down the lipidic biosynthetic pathways [116]. One-carbon metabolism, a pathway to provide methyl groups for various biological reactions (e.g., nucleic acids including DNA, amino acids and phospholipids), plays key roles in cellular homeostasis and epigenetic regulations, along with the closely linked pathways (e.g., trans-sulfuration and redox control). Dysregulation of these pathways has been implicated in the pathogenesis of NAFLD [117,118,119]. In humans, paraoxonase-1 serum concentration was significantly reduced in patients with NAFLD compared to controls and *PON1* L55M polymorphism was a significant predictor of NAFLD [120]. Further, serum homocysteine levels were significantly higher in patients with NAFLD compared to controls and Methylenetetrahydrofolate Reductase (MTHFR) C677T homozygotes, but not heterozygotes, were more prevalent in patients with NAFLD [121,122]. Although the importance of one-carbon metabolism and its related pathways has been suggested in human NAFLD, the significance of one-carbon metabolism in NAFLD and NASH progression have not been investigated considering the important sex differences in one-carbon metabolism [123]. Glutaminolysis and branched chain amino acids have also been linked to NAFLD and NASH progression [124,125]. The branched chain amino acid pathway also exhibits sex differences [126], which further underscore the importance of rigorous analytic approaches considering sex differences and menopausal status in the pathogenesis.

#### 3.3.6. Obesity

Obesity, an established risk factor for NAFLD, exhibits a sexually dimorphic pathophysiological profile. Men and women have distinct regional fat distribution and adipocyte biology, influenced by sex hormones. In obese individuals, the infusion of lipids induces a similar inhibition of glucose disposal in obese women and men; compared to obese males, however, obese females are more insulin-sensitive [127]. Such a difference is not accounted for in the variability in the intramuscular concentration of lipid intermediates, such as ceramide and diacylglycerol [127]. Ter Horst et al. investigated the differences in glucose metabolism between BMI-matched severely obese men and women using tissue-specific measurements of insulin sensitivity assessed with a two-step euglycemic hyperinsulinemic clamp with infusion of [6,6-2H2] glucose. Data have shown that, compared to obese women with similar BMI, obese men have lower hepatic insulin sensitivity which may contribute to the higher prevalence of diabetes in obese men; however, irrespective of sex, insulin sensitivity in adipose tissue and peripheral tissue was comparable among these obese individuals [128], which partly contradicts the above-mentioned findings from the study of non-obese Singaporeans [102].

#### 3.3.7. Aging

Aging or accelerated cellular senescence is another risk factor for the development of NAFLD which may be interpreted under the perspective of sex-dimorphic pathophysiology. Women are in general more sensitive to insulin compared to men. Studies conducted in humans have shown that the aging-related decline in insulin sensitivity is more pronounced in females although peripheral insulin sensitivity decreased with aging in both sexes. In the group with age < 40 years, females had significantly higher insulin sensitivity and lower insulin-receptor binding as compared to males. In those aged > 40 years, females still had higher peripheral insulin sensitivity but also higher insulin-receptor binding than men. With aging, the percentage of specifically receptor-bound insulin increased in females but decreased in males. As reproductive status was not assessed in the study, whether the observed sex differences are related to differences in physiological sex hormone levels is not answered. A higher androgen level in males affects the post-receptor processes in insulin action, which may account for the finding that males have lower insulin sensitivity, despite the higher insulin-receptor binding [129]. These findings imply that, in the analysis of peripheral insulin effectiveness and insulin-receptor binding, both gender and age should always be taken into account.

## 4. Sex-Differences in Relevant Co-Morbidities and Complications Observed in NAFLD

### 4.1. Type 2 Diabetes

T2D is both a cause and an effect of NAFLD [86,130]. Sex differences in these associations are clinically relevant.

### 4.2. From NAFLD to T2D

The risk of developing T2D differs between men and women [86,131]. There are a few clinical studies addressing the development of DM in patients with NAFLD, although data should be cautiously interpreted. By following 402 Japanese patients with biopsy-proven NAFLD for 4.2 years, Akuta et al. showed that female sex was a strong and independent risk factor for the development of incident T2D Hazard Ratio (HR 5.83, 95% confidence interval (CI) = 1.47–23.1, *p* = 0.012) [132]. In the study, subjects with T2D at the time of liver biopsy were excluded from the analysis. Thus, the observed female dominance in the DM development may reflect the increased risk of DM in women after menopause. Although this study is amenable to criticism based on methodological grounds, the finding appears to be in substantial agreement with a previous study which has shown that the incidence of T2D is higher in post-menopausal women with hepatocyte steatosis [133]. Along the same line, another study found that the combination of fatty liver and insulin resistance is a stronger risk factor of diabetes in women as compared to men (6x versus 4x) [134]. These data may collectively pave the way to further research being conducted aimed at exploring the role of NAFLD in the development of postmenopausal T2D.

### 4.3. From T2D to NAFLD 

Data regarding sex differences in the prevalence of NAFLD among those with T2D appear to be conflicting. Wild et al. showed that, by linking the population-based Scottish diabetes registry to hospital, cancer and death records, 6667 versus 33,624 incident chronic liver disease were identified in people with and without T2D respectively, after following over 1.8 and 24 million person-years and, among them, the risk of NAFLD in T2D were significantly higher in women than men [135]. In contrast, Dai et al. showed in their meta-analysis (35,599 T2D patients and 20,264 with NAFLD) that the prevalence of NAFLD was in the same order of magnitude in both sexes (60.11% (CI 53.63–66.41) for men and 59.35 (CI 53.28–65.28) for women) [136].

### 4.4. Uric Acid

Increased serum uric acid (SUA) levels are also a risk factor for NAFLD not only among Asians but also in western populations [137,138]. Interestingly, Fan et al., by analyzing data in 541 patients with T2D, found that elevated levels of SUA were a risk factor for NAFLD only in men but not in post-menopausal women, and suggested that this association—independent of insulin resistance and other metabolic confounding factors—is specific for men with T2D [139]. Conflicting with this study, however, Hwang showed that, in 9019 Koreans undergoing a health check-up, subjects with the highest SUA quartile were associated with a higher risk of NAFLD compared to subjects with the lowest SUA quartile, regardless of gender, but the effect of SUA was higher in women (OR 2.13 (95% CI 1.42–3.18)) than in men (OR = 1.46 (95% CI 1.17–1.82)) [140]. The inconsistent results may partly be explained by age difference (and the lack of the consideration of menopausal status) in the study population. The average age of these individuals was younger in the study by Hwang.

### 4.5. Atherosclerosis

Female sex is protective from cardiovascular disease (CVD) [61]. Atherosclerosis and related cardiac diseases are the leading causes of death among those with NAFLD [85,141]. Men with NAFLD are particularly prone to this risk. A recent large study, evaluating 603 patients with biopsy proven NAFLD who were free of CVD at baseline and 6269 population controls, found that during a mean follow-up of 18.6 years, male sex was an independent predictor of CVD events among those with NAFLD. However, the diagnosis of NASH or the fibrosis stage did not show association with the CVD events. [142]. Of interest, women with NAFLD lose the CVD protection conferred by the female sex. In a recent cohort study done in Olmsted County, Minnesota, USA, in which 3869 patients with NAFLD and 15,209 age- and sex-matched subjects were followed for 7 years (range 1–20), women were protected from ischemic CV events in the general population (HR = 0.71, 95% confidence interval 0.62–0.80, *p* < 0.001), even after stratification by personal history of CVD, BMI at baseline and time-dependent smoking, diabetes, arterial hypertension, dyslipidemia and variables indicating access to medical care, but not among patients with NAFLD, the protection was eliminated (HR = 0.90, 95% CI 0.74–1.08, *p* = 0.25). Of concern, excess CVD events and mortality as compared to age- and sex-matched referents were higher in women with NAFLD than in men with NAFLD [143].

### 4.6. Arterial Hypertension and Chronic Kidney Disease (CKD) 

Whether sex/gender also modulates the risk of developing arterial hypertension and CKD in those with NAFLD remains to be investigated. A study conducted in 1006 Chinese adults showed that the prevalence of hypertension and NAFLD were significantly higher in men than in women (*p* < 0.05); however, after adjusting for potential confounders, NAFLD was associated with an increased risk of hypertension in both men and women with a similar effect (OR = 2.152 (95% CI) (1.324–3.498) and OR = 2.133 (95% CI) (1.409–3.229)), respectively [144].

Another study conducted in 455 consecutive adult primary solitary liver transplantation recipients found that female sex (OR 2.52, (1.25–4.71), *p* = 0.004), age (OR 1.05, (1.02–1.08), *p* = 0.003), and NASH (OR 2.95, (1.06–8.21), *p* = 0.039) were independent predictors of ≥stage 3 CKD during the 5-year follow-up after liver transplantation. Of note, different immunosuppressant use was univariately associated with the one-year outcome but not the five-year outcome [145].

### 4.7. Hepatic Fibrosis

Given that liver fibrosis is a major determinant of hepatic and extra-hepatic mortality, answering the question as to whether the risk of fibrosis in NAFLD is a sex-dependent-phenomenon or not is key. A large body of literature supports the notion that estrogen, by activating ER-beta, inhibits hepatic fibrosis by inhibiting the activation and the proliferation of hepatic stellate cells [146,147,148]. In agreement, supporting previous epidemiological studies [46,50], a recent study confirms that women may be spared from advanced fibrosis. Tobari et al., by evaluating a large series of 762 patients (53% men) with biopsy proven NAFLD, found that advanced fibrosis was significantly more common among severely obese men than among severely obese women (*p* < 0.01) [149]. Machado et al., who, by evaluating 1620 patients with severe obesity in twelve observational and transversal studies, found that male sex was associated with NASH/hepatic fibrosis [150]. These findings are consistent with other clinical and translational studies [42,43]. How can these findings be explained? Further to the previously cited estrogen’s protective effect on stellate cell activation and fibrogenesis, Marcos et al. [151] have astutely highlighted that the liver histology is sexually dimorphic. In the normal livers of male rats, collagen tends to be more abundant compared to the “female liver”. Conversely, female rats have more hepatocytes and these cells have more membrane fluidity and Kuppfer cells than males [151]. Whether such a sexual dimorphism is observed in human normal livers remains to be fully investigated but may contribute to sex differences in the pathology of NAFLD liver.

### 4.8. Sub-Acute, Progressive Presentation of NASH 

It has been known for a long time that NASH-cirrhosis can have a sub-acute course and develop into liver failure with unknown mechanisms [152]. Table 3 summarizes the principal reports illustrating this rare, dramatic occurrence in the natural history of NAFLD [153,154,155].

As shown in Table 3, women are over-represented among these cases. The reason for the female dominance in this particular phenotype is unknown. Further case collection and characterization are necessary to ascertain the female dominance in this dangerous outcome and investigate those underlying mechanisms. 

### 4.9. HCC and Other Benign and Malignant Tumors

NAFLD is increasingly associated with a variety of (hepatic and extra-hepatic) cancers [156,157], and the association exhibits sex-specific patterns. Men with NAFLD are at a high risk of prostate cancer versus control men, particularly in the elderly, even in the absence of obesity or MetS [158], and HCC in those with cirrhosis, irrespective of its etiology [159]. Moreover, according to a study on 3686 individuals (2430 males and 1256 females) who had health checkups, NAFLD was associated with an increased risk of colorectal adenomatous and hyperplastic polyps in men but not in women [160]. Kwak et al. found, in a case-control study including 270 breast cancer cases, that NAFLD was significantly associated with breast cancer at multivariate analysis (*p* = 0.046), independent of traditional risk factors. Interestingly, this association was observed in the non-obese subgroup but not in the obese subgroup [161].

Sex differences in inflammatory cytokines and immune response may be one of the underlying reasons accounting for these sex differences in cancer risk under metabolic stress [162]. Further studies are warranted to investigate tumorigenesis pathways while considering sex differences.

NAFLD exhibits an increased risk of developing HCC in the absence of cirrhosis [163,164,165]. The incidence of HCC in non-cirrhotic NAFLD is about a tenth of the HCC risk in cirrhotic patients [166,167]; therefore, HCC surveillance is not cost-efficient in non-cirrhotic NAFLD [168]. Tobari et al., by comparing non-cirrhotic NAFLD patients with or without HCC (*n* = 612), showed that male gender (OR: 7.774, 95% CI: 2.176–27.775), light drinking (OR: 4.893, 95% CI: 1.923–12.449) and high FIB4 index (OR 2.634, 95% CI: 1.787–3.884) were independent predictors of HCC. They also demonstrated that the male dominance was more prominent among non-cirrhotic HCC compared with cirrhotic HCC [169].

## 5. Implications for Practice: Should Sex-Specific Lifestyle Changes Be Adopted?

Whether or not lifestyle modifications (as well as drug therapy) should be differentially employed based on the individual patient’s sex or menopausal status is a key research question still in need of evidence-based answers. Preclinical studies provide robust evidence to support that males and females respond differently to caloric restriction, intermittent fasting and keto-diets [170,171], but robust human data are still lacking. In general, men lose more weight and present more metabolic benefits than women [172]. The greater metabolic benefits may be partly explained by the fact that men lose more visceral adiposity than subcutaneous adiposity during weight loss than women [173,174]. Similarly, in NAFLD patients, greater histologic improvement was observed in men than women after weight loss. Male sex is one of the factors predicting beneficial histological changes in response to a relatively modest weight loss (between 7% and 10%), while in women, a more substantial weight loss (>10%) is required to produce a significant histological improvement [175].

Physiological response to exercise appears to be different in men and women. Women’s muscle fibers differ from men’s [176]. Women have more type I muscle fibers than men [177,178], which have higher capabilities of lipid oxidation (e.g., greater lipid uptake, storage and oxidation) than type II fibers [179]. Type I muscle fibers also contain 2–3 times greater lipid content than type II fibers but are more sensitive to insulin actions [180], which may partly explain the fact that women’s muscles have a higher content of intramyocellular lipids than men’s muscle but are more sensitive to insulin. Despite the higher intramyocellular lipids in women, women have smaller intramyocellular lipid droplets than men, which may also explain metabolic advantages observed in women (e.g., higher capacity to oxidize lipid due to a higher lipid droplet surface area to volume ratio [180]).

Due to the above differences in muscles, women rely more than men on intramyocellular lipids as an energy source via the up-regulation of intramuscular lipid oxidization during exercise [176]. Further effects of exercise on resting lipid metabolism are sexually dimorphic [181]. Thus, optimal therapeutic exercise for weight loss and metabolic benefits (i.e., type, length and frequency) may differ between men and women, or between pre- and post-menopausal women. Such important questions should be addressed in future interventional studies.

Lastly, there may be gender differences in lifestyles. Inadequate physical activities and sedentary lifestyle are risk factors for NAFLD [182]. In the large epidemiological study, women showed a lower tendency of meeting the physical activity guidelines and showed a stronger beneficial effect of increased physical activity against NAFLD compared with men. Which gender-related factors (i.e., socio-cultural attributes) are associated with the lower physical activity among women remain unknown. Gender-specific socio-cultural factors affecting the risk of NAFLD warrant future investigation. 

## 6. Conclusions

As we discussed, there is robust evidence for sex differences in NAFLD and relevant clinical conditions (e.g., insulin resistance, other metabolic disorders, CVD). However, as we reiterated above, significant gaps still exist to develop sex-specific clinical guidelines and management. Genetic and epigenetic studies can provide mechanistic insights into NAFLD pathobiology, information that can improve disease staging, risk assessment and prediction of disease progression and complications (such as cirrhosis and HCC). However, currently available data in the literature lack the consideration of sex/gender and menopausal status in the analysis, which may have undermined specific genetic factors that modulate the disease risk in a specific sub-population. Proper consideration of potential sex differences in future research, not just clinical/epidemiological studies, but also genetic analysis and clinical trials, is critical to advancing our knowledge to personalizing our practice.

Incorporating possible sex differences and sex/age interaction in study design and analytic strategy poses methodological challenges. Statistical assessment of interactions, in general, requires a larger study population. Including males and females in experiments increases the number of animals and the cost of the experiments. The assessment of sex differences, sex interactions and two-way interactions (sex and age) certainly complicates statistical analysis.

Given the robust evidence of sex differences in NAFLD pathobiology, however, such rigorous investigation is critical to building our knowledge of precision medicine further. In future clinical/epidemiological studies, sex-specific and sex/age-specific analyses should be performed, and sex and menopausal status should be collected when possible and considered as potential effect modifiers unless proven otherwise.

## Figures and Tables

**Table 1:** Sexual dimorphism of nonalcoholic fatty liver disease (NAFLD). Evidence from experimental studies.

Author, Year [Ref]	Method	Findings	Conclusions
Kirsch, 2003 [10]	Male and female Wistar, Long-Evans and Sprague-Dawley rats, and C57/BL6 mice (*n* = 6 per group) were fed a methionine-choline-deficient (MCD) diet for 4 weeks.	Compared to females, male rats fed the MCD diet developed greater steatosis (*p* < 0.001), had higher liver lipid content (*p* < 0.05) and had higher serum alanine transaminase (ALT) levels (*p* < 0.005). Compared to Wistar rats, male C57/BL6 mice exhibited more inflammatory foci (*p* < 0.001), end products of lipid peroxidation (*p* < 0.005) and mitochondrial injury, despite the fact that they had less steatosis (*p* < 0.005) and lower products of lipid peroxidation.	The Wistar strain and the male sex are associated with the greatest degree of steatosis in rats subjected to the MCD diet. Male C57/BL6 mice develop a histological picture more faithfully resembling NASH, with necro-inflammatory changes, lipid peroxidation and ultrastructural injury.
Stöppeler, 2013 [11]	Lewis and Sprague-Dawley rats of both sexes were fed either a standard or high-fat diet (HFD) for three weeks.	Female rats of both Lewis and Sprague-Dawley strains had lower steatosis grade, lower fibrosis and higher proliferation rate of non-parenchymal cells than males (*p*, 0.05). Lewis female rats on a HFD had lower serum alkaline phosphatase, cholesterol, triglyceride and leptin levels and a more favorable low-density/high-density (LDL/HDL) cholesterol ratio than males (*p* < 0.05). HFD induced downregulation of proangiogenic genes (*p* < 0.05) in males but not in females.	Together with strain, sex plays a major role in the development and progression of experimental NAFLD.
Chukijrungroat, 2017 [12]	Male, female and ovariectomized (OVX) Sprague-Dawley rats were fed either a control diet or HFD for 12 weeks.	HFD induced: A higher degree of hepatic steatosis, with significant increases in proteins involved in hepatic lipogenesis in females than males, Liver injury, inflammation and oxidative stress in males, but not females. A significant increase in hepatic FGF21 protein expression was found in HFD-fed males (but not in females). The deprivation of estrogen per se was associated with a significant reduction in fibroblast growth factor (FGF)21 with hepatic steatosis, and HFD further aggravated steatosis in OVX rats. Conversely, estrogen replacement reduced hepatic steatosis in HFD-fed OVX rats by restoring hepatic FGF21 levels.	Male rats are more susceptible to HFD-induced hepatitis, while females developed a higher degree of hepatic steatosis. The latter was associated with the hepatic expression level of FGF21.
Kurt, 2018 [13]	A comprehensive multi-omics approach, integrating genomics and transcriptomics of liver and adipose tissue with phenotypic data of hybrid mouse diversity panel (HMDP), was adopted. The NAFLD molecular pathways and gene networks were compared between sexes.	Adaptive immunity, branched chain amino acid metabolism, oxidative phosphorylation and cell cycle/apoptosis were shared between sexes. Vitamins’ and cofactors’ metabolism and ion channel transport were specific for females, and phospholipid, lysophospholipid and phosphatidylinositol metabolism and insulin signaling for males. Moreover, several pathways controlling lipid- and insulin-metabolism and inflammatory processes in the adipose and liver tissue were more prominently associated with NAFLD in male HMDP.	Both shared and sex-specific biological processes for NAFLD were identified. NAFLD pathways are regulated in a sex- and tissue-specific manner.
Camporez, 2019 [14]	Age-matched or body weight-matched female and male mice were fed an HFD or regular chow for four weeks. HFD-fed male mice were also treated with either estradiol or vehicle.	Compared to HFD-fed male mice, HFD-fed female mice and estradiol-administered HFD-fed male mice were associated with increased whole-body insulin sensitivity and decreased hepatic and muscle lipid content. The decreased ectopic lipid content in HFD-fed female mice and estradiol-administered HFD-fed male mice was associated with increased insulin-stimulated suppression of WAT lipolysis and decreased WAT inflammation.	Estradiol mediated reductions in WAT inflammation and subsequent increase in insulin-induced suppression of WAT lipolysis and reduced deposition of intra-hepatic and intra-muscular fat protects HFD-fed mice from insulin resistance (IR) associated with obesity and explains the protection from IR in females.
McCoin, 2019 [15]	Male and female wild-type (WT), liver-specific proliferator-activated receptor gamma coactivator 1 alpha (PGC-1α) heterozygote (LPGC-1α) and BNIP3 null mice were housed at thermoneutral conditions. Mice were then divided into three groups: sedentary-low-fat diet (LFD), 16 weeks of HFD, or 16 weeks of HFD with voluntary wheel running (VWR) for the final 8 weeks (HFD + VWR).	Females were associated with higher hepatic mitochondrial respiratory coupling control, lower mitochondrial respiratory H2O2 emission and were protected from steatosis and fibrosis compared with males in all conditions. VWR was required in male mice to elicit those mitochondrial adaptations. Steatosis and markers of liver injury, which occurred in sedentary male mice fed a HFD, were effectively reduced by VWR despite persistent hepatic steatosis. HFD + VWR significantly increased maximal respiratory capacity only in WT and PGC-1α females. Loss of BNIP-3-mediated mitophagy blunted these responses in females, while males only had a non-significant increase in mitochondrial respiration with HFD plus VWR.	Sex was the primary determinant for mitochondrial adaptations to a chronic HFD and HFD plus increased physical activities in the diet-induced fatty liver model. Effects of reduction/deficit in PGC-1α or BNIP3 on mitochondrial adaptation to HFD and increased physical activities significantly differ between males and females.

**Table 2:** Conflicting findings regarding sex as a risk factor for fibrosis in clinical observational studies.

Author	Study Population/Ethnicity. Menopausal Status	Diagnostic Criteria	Findings
**Risk of NASH and advanced fibrosis higher in women than in men**			
Miyaaki, 2008 [41]	182 Japanese (74 men and 108 women). Age 50.81 ± 15.22 years. Menopausal status not addressed (NA)	Ultrasonography. Exclusion of competing liver diseases. Liver histology	Female sex (*p* = 0.002), older age (≥60 years old) (*p* = 0.020), T2D (*p* = 0.020) and arterial hypertension (*p* = 0.002) were significantly associated with severe fibrosis at multivariate analysis.
Singh, 2008 [42]	71 Asian-Indians (54 men and 17 women). Age range 9–57 years. Menopausal status or pubertal stage NA	Liver histology	Female sex (*p* = 0.02), serum alkaline phosphatase (*p* = 0.018), total cholesterolemia (*p* = 0.048) and LDL serum cholesterol concentrations (*p* = 0.025) were independent predictors of the stage of fibrosis at multivariate analysis.
Bambha, 2012 [43]	628 USA adults (201 men and 427 women) with NASH out of a larger cohort of 1026 adults enrolled in the NASH Clinical Research Network (CRN) database from 2004 to 2008 (483 were non-Latino whites and 74 were Latinos, 28 Asians, 14 non-Latino Blacks, 29 other ethnicities). Aged 50 years (49.1–50.9). Menopausal status NA.	Liver histology	Age (Odds Ratio (OR) 1.02 Confidence Interval (C.I.) 1.01–1.04)); female sex (OR 1.59 (C.I. 1.04–2.41)); AST (OR 1.07 (C.I. 1.03–1.12)); ALT (OR 0.95 (C.I. 0.92–0.99)); Platelet count (OR 0.91 (C.I. 0.88–0.94)); arterial hypertension (OR 2.20 (C.I. 1.41–3.42)); Homeostasis Model Assessment (HOMA)-IR (OR 1.08 (C.I. 1.04–1.12)); Alkaline phosphatase (OR 1.09 (C.I. 1.03–1.15)) and total cholesterol (OR 0.94 (C.I. 0.89–0.98)) were associated with advanced fibrosis at multivariate logistic regression analysis.
Tapper, 2014 [44]	358 individuals (225 men, of whom 149 were Caucasians, and 133 Women, 108 of whom were Caucasians). Mean age: men 45.3 ± 11.2 years, women 51.4 ± 10.6 years. Menopausal status NA.	Ultrasonography. Liver histology	Female sex (OR 1.76 (C.I. 1.01–3.10)), BMI ≥ 30 kg/m^2^ (OR 2.21 (C.I. 1.23–4.08)) and AST (>40 IU/L) (OR 2.00 (C.I. 1.14–3.55)) were the independent predictors of advanced NASH (NAFLD Activiy Score (NAS) >4)) at multivariate analysis.
**Risk of NASH and advanced fibrosis higher in men than in women**			
Hossain, 2009 [45]	432 USA patients. Male 22.86%: Age 43.6 ± 11.4 years. Menopausal status NA.	Liver histology	The multivariate analysis model used to predict moderate-to-severe fibrosis included male sex, Caucasian ethnicity, T2D and increased aspartate transaminase (AST) and ALT levels (*p* < 0.0001).
**Risk of NASH and advanced fibrosis depends on both sex and age (e.g., menopausal status)**			
Yang, 2014 [46]	Cross-sectional study conducted on 541 adults with biopsy proven NASH. Menopausal state and synthetic hormone use were identified based on self-reported information. Men (average age 45.9 ± 11.7 years), pre-menopausal women (average age 40.1 ± 7.8 years) and post-menopausal women 56.0 ± 7.4 years) comprised 35%, 28% and 37% of the study population, respectively.	Liver histology	After adjusting for covariates (enrolling site, grades of portal inflammation and hepatocyte ballooning) and potential confounders (race, body mass index, diabetes/pre-diabetes, hypertension), adjusted cumulative OR (ACOR) and 95% confidence interval (CI) for greater fibrosis severity was 1.4 (0.9, 2.1) (*p* = 0.17) for post-menopausal women and 1.6 (1.0, 2.5) (*p* = 0.03) for men, having pre-menopausal women as a reference. After adjusting for the covariates (the site of enrollment, grade of portal inflammation and hepatocyte ballooning), estrogen replacement was associated with a 50% risk reduction, although the association did not reach statistical significance (ACOR = 0.5; 95% CI: 0.2-1.2; *p* = 0.11).
**No sex differences in risk of NASH and advanced fibrosis**			
Yatsuji, 2007 [47]	193 biopsy proven NASH patients (86 women and 107 men) with mean age of 53 years in Japan.	Liver histology	Overall, 38.3% showed advanced fibrosis (23.8% under 55 years old, 54.3% 55 years or older). Among patients with advanced fibrosis (stage 3 or 4), men were more prevalent in the younger group (75%), while, in the older group, women were more prevalent (66%). The gender difference in advanced fibrosis was not statistically significant. In a multivariable model, older age and BMI were significantly associated with advanced fibrosis in the younger group. In the older group, the lack of hyperlipidemia was associated with advanced fibrosis.
Labenz, 2018 [48]	261 patients with biopsy proven non-cirrhotic NAFLD (mean age 51, ranging from 19 to 93, with equal sex distribution) in Germany.	Liver histology	15.7% showed advanced fibrosis (stage 3). Patients with advanced fibrosis were older, had higher BMI and a higher prevalence of hypertension and type 2 diabetes. Gender was not associated with advanced fibrosis. In a multivariable model, hypertension and type 2 diabetes were significantly associated with advanced fibrosis.

**Table 3:** Fulminant liver failure and hyper-acute NASH.

Author, Year [Ref]	Method	Subacute Liver Failure/Hyper-Acute NASH	Conclusions
Caldwell, 2002 [153]	Out of a cohort of 2380 patients with liver disease, 167 had NASH and 215 had cryptogenic cirrhosis. Five cases followed a sub-acute course.	In 5 middle-aged obese women (one had T2D and one had glucose intolerance), their previously unrecognized liver disease followed a sub-acute course and, over a 4–16 week period, 4 died of liver failure and the fifth patient underwent ortotopic liver transplantation (OLT). Histology revealed previously unrecognized cirrhosis with ballooned hepatocytes in all 5 patients, frank steatohepatitis in 3, necrosis in 2 and micro-macrovesicular steatosis in 1.	These obese and middle-aged women had previously unrecognized cirrhosis and sudden deterioration of uncertain cause. Clinical and histological findings support the notion that these ladies had undiagnosed NASH, silent progression to cirrhosis and, finally, subacute liver failure.
Kranidiotis, [154]	Case-report	A 50-year old man with MetS experienced acute liver failure as the first manifestation of NASH. The patient was submitted to be insulin treated. This, together with lifestyle changes, resulted into a dramatic improvement of clinical and laboratory data.	
Tsai, 2017 [155]	All cases suggesting rapidly progressing liver disease were retrieved by searching for ‘steatohepatitis.’ files from 2000 to 2015. Criteria for exclusion: any history of alcohol or drug abuse. One case was received from another Institution.	In 6 women with a median age of 39 years, with no history of excess alcohol consumption nor of prior liver disease, severe hepatic failure was observed after rapid weight loss (18 to 91 kg) following Roux-en-Y gastric bypass (in 4 patients) and starvation-like dieting or hypoalbuminemia (in 2 patients). Four patients either died or were submitted to OLT. Liver histology disclosed severe steatohepatitis, including extensive/circumferential centrizonal pericellular fibrosis, central scar with perivenular sclerosis/veno-occlusion with superimposed hepatocellular dropout, abundant/prominent hepatocellular balloons and numerous Mallory–Denk bodies.	Aggressive NASH is associated with rapid weight loss/malnutrition. While it may not be possible to entirely exclude an alcohol etiology, the observed histological findings should not specifically be attributed to alcohol and may represent an acute to sub-acute response to central zone injury during rapid weight loss/malnutrition. Histological findings pointing to a similarity of histogenic and pathogenic mechanisms in alcoholic and NASH.

## Abbreviations

ACOR	adjusted cumulative odds ratio
ALT	alanine transaminase
AMPK	adenosine monophosphate kinase
AST	aspartate transaminase
BAA	BMI, age, ALT and TG
BMI	body mass index
CKD	chronic kidney disease
CI	95% confidence interval
CRN	Clinical Research Network
CRP	C-reactive protein
CT	computed tomography
CVD	cardiovascular disease
E2	17β estradiol
ER	Estrogen Receptors
FGF21	fibroblast growth factor 21
Fib4	fibrosis 4
GGT	gamma-glutamyl transferase
GPER	G-protein-coupled Estrogen Receptor
HCC	hepatocellular carcinoma
HFD	high-fat diet
HMDP	hybrid mouse diversity panel
HR	hazard ratio
MCD	methionine and choline deficient
NA	not addressed
NAFLD	nonalcoholic fatty liver disease
NASH	nonalcoholic steatohepatitis
NEFA	non-esterified fatty acids
OLT	ortotopic liver transplantation
OR	odds ratio
OVX	ovariectomized
PAT	pericardial adipose tissue
PGC-1α	proliferator-activated receptor gamma coactivator 1 alpha
PIVENS	Pioglitazone versus Vitamin E versus Placebo for the Treatment of Non-diabetic Patients with Nonalcoholic Steatohepatitis
STD	standard
TG	triglycerides
T2D	type 2 diabetes
VWR	voluntary wheel running
